# Blood proteome profiling using aptamer-based technology for rejection biomarker discovery in transplantation

**DOI:** 10.1038/s41597-019-0324-y

**Published:** 2019-12-09

**Authors:** Andrey V. Shubin, Branislav Kollar, Simon T. Dillon, Bohdan Pomahac, Towia A. Libermann, Leonardo V. Riella

**Affiliations:** 1 Department of Molecular and Cellular Biology, Harvard University, Cambridge, MA 02138 USA; 2 Division of Plastic Surgery, Department of Surgery, Brigham & Women’s Hospital, Harvard Medical School, Boston, MA 02115 USA; 3 Beth Israel Deaconess Medical Center Genomics, Proteomics, Bioinformatics and Systems Biology Center, Division of Interdisciplinary Medicine and Biotechnology, Beth Israel Deaconess Medical Center, Harvard Medical School, Boston, MA 02215 USA; 4 Schuster Transplantation Research Center, Renal Division, Brigham & Women’s Hospital, Harvard Medical School, Boston, MA 02115 USA

**Keywords:** Diagnostic markers, Risk factors, Proteomic analysis, Transplantation tolerance

## Abstract

Face transplantation is a promising solution for patients with devastating facial injuries who lack other satisfactory treatment options. At the same time, this type of transplantation is accompanied with high risks of acute transplant rejection. The limitations of traditional skin biopsy and the need to frequently monitor the condition of face transplant call for less invasive biomarkers to better diagnose and treat acute rejection. Discovery of peripheral serum proteins accurately reflecting the transplant status would represent a reasonable solution to meet this demand. However, to date, there is no clinical data available to address the feasibility of this approach. In this study, we used the next generation aptamer-based SOMAscan proteomics platform to profile 1305 proteins of peripheral blood serum in twenty-four samples taken from 6 patients during no-rejection, nonsevere rejection, and severe rejection episodes. Also, we provide a detailed description of biosample processing and all steps to generate and analyze the SOMAscan dataset with hope it will assist in performing biomarker discovery in other transplantation centers using this platform.

## Abstract (summary)

**Table:** 

Measurement(s)	biomarker • graft rejection AE
Technology Type(s)	proteomics platform
Factor Type(s)	transplant rejection severity
Sample Characteristic - Organism	Homo sapiens

Machine-accessible metadata file describing the reported data: 10.6084/m9.figshare.10548122

## Background and Summary

Reconstructive transplantation such as face transplantation (FT) is a promising way to restore the functionality and social reintegration for severely disfigured patients^1,2^. More than forty such procedures have been performed world-wide to date^3^. However, face transplantation is traditionally associated with almost 6-fold higher rates of acute rejection as compared to solid organ transplantation^4^. Acute rejection in FT is usually diagnosed by clinical inspection of the transplanted face together with histopathological skin biopsy assessment according to Banff grading^5,6^. Unfortunately, both modalities are associated with limitations such as intra- and inter-observer variability, interchangeability with other non-alloimmune conditions and more importantly, lacking correlation to treatment response^7,8^. Therefore, there is an unmet need for improved immune monitoring tools. Previous studies examined biomarkers of rejection from skin biopsy samples in both experimental and clinical setting^9–13^. However, there is almost no data about the utility of non-invasive biomarkers to help diagnose rejection in FT. To bridge this gap, we used an aptamer-based platform SOMAscan to discover acute rejection biomarkers from blood of six face transplant recipients^14^.

**Fig. 1:**
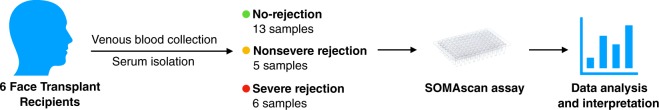
Scheme of the study design. 24 serum samples from all 6 face transplant patients representing no-rejection (n = 13), nonsevere rejection (n = 5) and severe rejection (n = 6) were included into the SOMAscan analysis. Severe rejection episodes required steroid bolus or other more potent drugs for resolution. Nonsevere rejection episodes were reversed by maintenance immunosuppression adjustment and/or topical therapy only.

SOMAscan allows a multiplexed assessment of 1,305 proteins from as little as 50 μL serum sample with high sensitivity (38 fM) and a broad dynamic range (>8 logs)^15,16^. The utility of SOMAscan has been previously demonstrated in various settings such as of cardiovascular disease^17,18^ and cancer^19,20^ but not much is known about how it performs in transplant-related biomarker discovery. Our study was the first blood proteome characterization in face transplantation using longitudinal serum samples from six face transplant patients^14^. In this retrospective study, we included twenty-four serum samples from 13 no-rejection, 5 nonsevere rejection and 6 severe rejection episodes for biomarker discovery using the SOMAscan assay (Fig. 1). This sample division was based on the treatment response: nonsevere rejections resolved with topical therapy and/or maintenance immunosuppression adjustment while severe rejections necessitated systemic administration of glucocorticoids or more potent immunosuppressive drugs. This methodology has high clinical relevance because the treatment of nonsevere rejections is associated with lower patient morbidity. Furthermore, the current diagnosis of acute rejection based on skin biopsy and clinical presentation partially fails to distinguish nonsevere from severe rejections. Finally, to test reproducibility of SOMAscan results, MMP3 as a promising candidate of severe rejection was technically validated with a gold-standard immunoassay^14^.

Here we provide a detailed description of steps which were involved in biosample processing and then generation and analysis of the SOMAscan dataset. We believe that by sharing this information, we can help motivate and guide other transplantation centers on how to perform biomarker discovery using this platform. Further, we aim to provide fundament for standardization and integration of our data into larger multicenter meta-studies, which will ultimately advance this field.

## Methods

### Study approval

Written informed consent for participation in the clinical trial (ClinicalTrials.gov number NCT01281267) and for collection and processing of their blood samples (approved by the IRB at Brigham and Women’s Hospital, Protocol #: 2010P000743) was obtained from all 6 patients. The clinical trial was approved by the institutional review board (IRB) at Brigham and Women’s Hospital (Protocol #: 2008BP00055).

### Patients, immunosuppression and treatment of rejection

Six patients received face transplants that were matched according to sex, skin color and ABO compatibility (Table 1). To minimize the risk of hyperacute transplant rejection, all patients had negative T- and B-cell cytotoxic crossmatch, with exception of one highly pre-sensitized patient (Patient 4) with a weakly positive cytotoxic T-cell crossmatch (20%)^21^.

**Table 1:** Patients’ characteristics.

	Patient 1	Patient 2	Patient 3	Patient 4	Patient 5	Patient 6
Date of transplant	05/2011	03/2011	04/2011	02/2013	03/2014	10/2014
Age at transplant (years)	57	25	30	44	38	33
Gender	F	M	M	F	M	M
Ethnicity	White	White	White	White	White	White
Mechanism of injury	Animal Attack	Electrical Burn	Electrical Burn	Chemical Burn	Ballistic trauma	Ballistic trauma
Graft type	Full Face, Bilateral Hands	Full Face	Full Face	Full Face	Partial Face	Partial Face
Ischemia time (hours)	2	4	2	3	3	1.5
PRA (%)	0	68	0	97	22	32
DSA	Negative	Negative	Negative	Positive	Negative	Positive
HLA mismatch (A, B, C, DR, DQ, DP)	8	8	5	11	8	7
CMV (Donor/Recipient)	Positive/Positive	Positive/Positive	Positive/Negative	Negative/Positive	Positive/Negative	Negative/Positive
EBV (Donor/Recipient)	Positive/Positive	Positive/Positive	Positive/Positive	Positive/Positive	Positive/Positive	Positive/Positive

The bilateral hands in Patient 1 were removed due to infectious complications in the first postoperative week. DSA, donor specific antibody; PRA, panel reactive antibody.

The induction therapy consisted of mycophenolate mofetil (1 g), methylprednisolone (500 mg), and rabbit anti-thymoglobulin (1.5 mg/kg/day for 4 days). Maintenance immunosuppression comprised typically triple therapy of tacrolimus (target levels of 8–12 ng/mL), mycophenolate mofetil (1 g twice daily) and prednisone. From postoperative month 14, patient 5 received belatacept in addition to the triple therapy. Steroids were completely weaned in 4 patients but reintroduced in 3 patients at 5 years of follow-up^2^.

Face allograft biopsies were performed at 3, 6, 12 months and then yearly as well as during suspected rejection (e.g., erythema, edema, exanthema). Acute cellular rejection was diagnosed from 4-mm skin punch biopsies according to the Banff classification of skin-containing composite tissues with five grades (Grades 0–IV)^5^. Only biopsies with grades equal or higher than II were considered for acute rejection therapy (Table 2) because mild non-specific inflammation resembling Grade I rejection is a common finding in healthy facial skin.

**Table 2:** Patients’ serum samples.

Patient	Posttransplant month	Histological Banff grade	Status	Clinical presentation	Rejection management
Patient 1	12	I	NR	NA	NA
Patient 1	17	II	NSR	erythema and edema, mucosa lesion	maintenance immunosuppression adjustment, topical therapy
Patient 1	24	0	NR	NA	NA
Patient 1	30	III	NSR	subclinical	maintenance immunosuppression adjustment
Patient 1	42	I	NR	NA	NA
Patient 2	18	0	NR	NA	NA
Patient 2	23	II	SR	erythema and edema	steroid bolus
Patient 2	24	III	SR	erythema and edema	ATG
Patient 2	48	III	SR	erythema and edema	steroid bolus
Patient 2	54	I	NR	NA	NA
Patient 3	12	0	NR	NA	NA
Patient 3	18	III	SR	exanthema	steroid bolus
Patient 3	34	III	SR	erythema	steroid bolus
Patient 3	54	0	NR	NA	NA
Patient 4	6	I	NR	NA	NA
Patient 4	13	III	NSR	erythema and edema	topical therapy
Patient 4	18	0	NR	NA	NA
Patient 4	24	III	NSR	erythema and edema	topical therapy
Patient 5	6	I	NR	NA	NA
Patient 5	7	III	SR	erythema and edema	steroid bolus, ATG, IVIG
Patient 5	9	I	NR	NA	NA
Patient 5	12	III	NSR	hyperpigmentation	maintenance immunosuppression adjustment, topical therapy
Patient 6	3	II	NR	NA	NA
Patient 6	12	0	NR	NA	NA

All rejection samples were from cellular-mediated rejections. ATG, anti-thymoglobulin; IVIG, intravenous immunoglobulin; NA, not applicable; NR, no-rejection; NSR, nonsevere rejection; SR, severe rejection.

Acute cellular rejection was treated by one or a combination of these modalities: steroid bolus (intravenous methylprednisolone 500 mg daily for 3 days followed by a taper), adjustment of maintenance immunosuppression or topical therapy with either steroids or tacrolimus. In case of steroid refractory rejections, anti-thymocyte globulin was administered.

More details about the patients, immunosuppressive protocols and rejection treatment can be found in previous publications^14,22,23^.

### Serum sample collection

Venous blood samples (approximately 10 mL) were prospectively collected in Red-Top (no anticoagulant) tubes (BD Vacutainer, Franklin Lakes, NJ) from transplant recipients at the following time points: pre-transplantation and post-transplantation at 24 hours, 1 week, 3, 6, 12 months, followed by six-monthly intervals; and during suspected rejection (Table 2). In case of suspected rejection, the samples were taken before therapy was initiated. To isolate blood serum, the tubes were centrifuged at 2,500 rpm for 15 mins. The supernatant was then transferred evenly into three 2 mL screw-cap cryovial-tubes (Corning, Fischer Scientific, Pittsburgh, PA). The serum was subsequently stored at −80 °C in the tissue repository where it could be retrospectively accessed for the proteomic analysis.

### SOMAscan assay

**Fig. 2:**
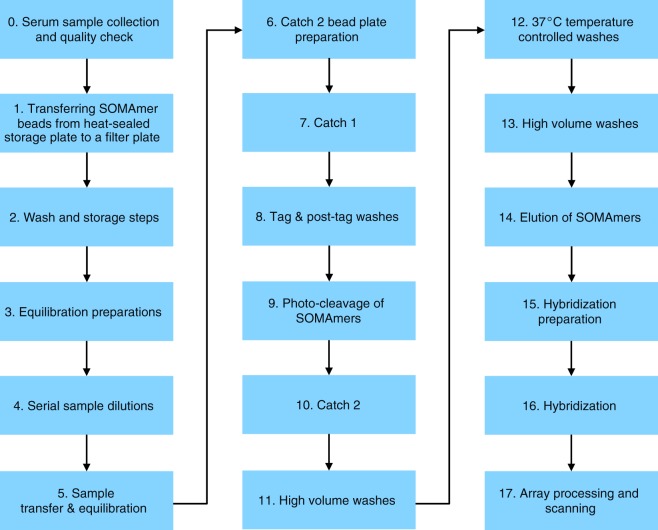
Steps of the SOMAscan assay.

SOMAscan analysis (SomaLogic; Boulder, CO) was performed at the BIDMC Genomics, Proteomics, Bioinformatics and Systems Biology Center using the SOMAscan Assay Kit for human serum, 1.3 k (cat. #900–00012, Supplemental Table S1), according to the standard protocol for serum from SomaLogic, as described previously^14^ (Fig. 2). Kit provided pooled human serum controls (five replicates) and one no-protein buffer control were run in parallel with the serum samples.

### Sample handling recommendations prior to assay

The first step prior to initiating the SOMAscan assay is the inspection of the serum samples for significant hemolysis. Lysis of red blood cells in a sample may be an indication that processing for that sample was not performed in a consistent or timely manner. Consistent and timely processing of the blood for serum isolation improves the reliability of the SOMAscan assay^24^. Hemolysis negatively impacts assay performance and therefore each sample is visually inspected for red color of the serum. Non-hemolyzed serum samples exhibit a translucent yellow color, samples with mild hemolysis are pink or (in the case of pronounced hemolysis) red. Strongly hemolyzed serum samples can lead to unreliable SOMAscan data and should be avoided as much as possible. The color of the serum sample should be noted and tracked with the sample. Our experience suggests that red samples frequently result in data indicating such samples as outliers, whereas pink samples typically do not pose a major confounding effect.

As sample processing and handling have significant effects on proteins and the SOMAmers recognize native proteins, various additional aspects should be considered when collecting, processing, storing, and handling serum samples. We have previously evaluated the impact of some of these aspects such as time of blood collection to plasma or serum processing on SOMAscan performance^25^ and noticed while time to processing did partially change the expression levels of proteins, a majority of the proteins were not significantly impacted. Similarly, freeze thaw cycles are known to impact proteins. Consequently, the number of freeze thaws should be noted and kept to only 2–3 cycles.

Immediately prior to analysis, serum samples should be thawed in a 25 °С water bath for 10 mins, then centrifuged for 10 mins at 14,000 g at room temperature to separate any debris, aggregates, and any other particulates. For example, excess lipid in the serum will be found at the top of the sample after this centrifugation step. Careful pipetting to avoid taking any of this lipid layer either within the pipet tip itself, or on the outside of the tip and transferring to the SOMAmer bead well is to be avoided. This is important in order to minimize and hopefully prevent clogging during the filtering steps of the assay.

### Preparations for assay

The manual version of the SOMAscan assay is described here, thirty-two samples including the kit controls are run at one time. Either 50 μL of serum is aliquoted directly into the first dilution on the day of the run, or the samples can be set up in a 96-well Nunc plate to be run on that day, or at a later time (in this case 65 μL are aliquoted). All samples, including the kit controls, are pipetted into their wells in the desired orientation. The plate is sealed with adhesive microplate foil sealing film (Thermo Scientific catalog #AB0626) and then stored in a tight fitting ziplock bag at −80 °С. Samples can be safely stored in this manner for up to one week. On the day of the assay the plate is carefully removed from the ziplock bag, a large KimWipe is pressed over the top of the foil sealing film to make sure it has not lifted off during −80 °С storage. The plate is gently floated onto room temperature deionized water in a shallow dish and allowed to thaw for approximately 15–20 mins. The sealed plate is dried off on the bottom and top, pressing down with a large KimWipe to make sure the foil is well attached and then centrifuged at 1000 g for 2 mins. The sealed top is carefully peeled off. Using a calibrated and well-maintained 8-channel multipipet set to 50 μL carefully and slowly pipet up and down 4–5 times to mix the samples, do not introduce any bubbles into the sample. 50 μL is then taken out to use in the assay for the 40% dilution. Visually check that each tip has the same volume by eye when transferring the removed samples to the first dilution (40% dilution as described in step 3 below). Filter tips are used throughout the assay to minimize the chance for cross contamination between the wells.

#### Transferring SOMAmer beads from heat-sealed storage plate to a filter plate

In the manual version of the assay the SOMAmer-Beads are provided in a Storage Plate which contains the biotinylated SOMAmers bound to Streptavidin-agarose beads. These plates can be stored when properly heat-sealed for up to three months at 4 °С with no degradation in performance. The Storage Plate is centrifuged for 2 min at 1000 g. The heat-sealed lid is then carefully removed. The SOMAmer-Beads from the Storage Plate are transferred in multiple wash steps to a filter plate (Millipore 0.45 µm hydrophilic, low protein binding membrane 96-well plate, catalog# MSHVN4510), named Catch 0 Filter Plate. Next, 150 μL SOMAmer-Bead Wash Buffer is transferred into the Storage plate and then removed to the 96 well assay filter plate. The filter plate is then subjected to vacuum filtration to remove the liquid. A multi-well plate vacuum manifold (Pall Corp., part# 5017) fitted with a custom 96-well vacuum plate wicker (SomaLogic, part# 400-00008) attached to house vacuum works well for the filtration steps. To capture more SOMAmer beads, 190 μL of the Bead Wash buffer is pipetted into the bead storage plate and then transferred to the filter plate. The filter plate is then shaken on an Eppendorf ThermoMixer C for 1 min at 25 °С at 850 rpm, followed by vacuum filtration to remove the buffer. This step is repeated three times to ensure that all the beads in the Bead Storage plate are transferred to the filter plate.

#### Wash and storage steps

The beads on the Catch 0 Filter Plate are washed twice with 190 μL Assay Buffer using the same ThermoMixer incubation, followed by vacuum filtration on the first wash and centrifugation for 2 min at 1000 g after the second wash step. Next, 190 μL Assay Buffer is added to the Catch 0 Filter Plate and a foil seal is carefully placed around the top perimeter of the plate. Use the flat top of a marker pen or similar device to press the foil on the top edges only, completely sealing the plate so it will not dry out. Avoid pushing hard on the foil over the wells or you will drive out liquid. The plate should be carefully balanced on the inverted top of the filter plate lid placed upside down. This activated SOMAmer bead filter plate can be stored at 4 °С for up to a week prior the actual running of the assay. Nothing should contact the bottom of the filter plate as this may wick liquid out of the wells.

#### Equilibration preparations

For SOMAscan to be able to achieve the ability to measure proteins across a very broad dynamic range the SOMAmers are separated into three separate bins for abundant, medium concentration, and low concentration serum proteins. To match these bins the serum sample is run at three unique dilutions. In order to accomplish this a carefully designed scheme of sample dilution has to be followed as described below.

In a 96-well Omni-Tube plate (ThermoScientific, part# AB-0407), 75 μL of serum diluent^16^ is transferred into wells A1 – H4 (for the undiluted serum to measure low abundance proteins, called the 40% bin), 195 μL Assay Buffer is pipetted into wells A5 – H8 for the 1% bin (1 to 40 dilution), and 133 μL is aliquoted into wells A9 – H12 for the 0,005% bin (1 to 8000 dilution for high abundance proteins). An additional 1:10 Dilution plate is prepared by loading 90 μL Assay Buffer into wells A9 – H12 of a second Omni-Tube plate for the 0.005% bin dilution.

#### Serial sample dilutions

If samples to be run were pre-aliquoted into a 96-well Nunc plate and stored frozen then this is thawed and spun down as described earlier in “Preparations for the Assay”. Next, 50 μL of the serum samples is transferred into wells A1 – H4 in the Sample plate (40% bin). Pipet up and down gently 5–10 times to mix well in the serum diluent. Then, transfer 5 μL from wells A1 – H4 into wells A5 – H8 (1% bin). Mix the 1% dilution well by using a multichannel pipettor set to 75 μL and gently pipetting up and down 5–10 times. To generate the 0.005% bin, 10 μL from wells A5 – H8 is first transferred to wells A9 – H12 in the 1:10 Omni dilution plate, again mix well by pipetting up and down with a multichannel set to 75 μL. To generate the final 0.005% subdilution 7 μL from wells A9 – H12 in the 1:10 Dilution plate are made to wells A9 – H12 in the Sample plate then gently mix up and down 75 μL. Cover the Omni plate containing the diluted samples with adhesive foil and centrifuge 1000 g for 2 min.

#### Sample transfer & equilibration

Centrifuge the Catch 0 Filter Plate on top of a 96 well collection plate at 1000 g for 2 min to remove the 0-AB storage liquid from the wells. Using an 8-channel pipette carefully transfer 100 μL from each column of the Samples Omni dilution plate into the Catch 0 Plate. Cover the Catch 0 Plate with adhesive foil, pressing down only on the edges to form a tight seal and avoid pushing the diluted sample liquid through the filter membrane. The plate is then carefully placed into the ThermoMixer C at 28 °С for 3.5 h for equilibration and binding of proteins to the SOMAmer beads. To prevent the sample wicking out the bottom of the filter plate, it should be held by the side metal pieces and not pushed to the bottom of the ThermoMixer C. The lid should be placed on the ThermoMixer C.

#### Catch 2 bead plate preparation

The next step is to prepare the magnetic beads for the second capture of biotinylated proteins bound to the eventually photocleaved SOMAmers. The bead storage buffer is removed from the magnetic beads first. Vortex the tube with the magnetic beads for 1 min using a standard benchtop Vortexer to make sure the beads are well suspended. Incubate the beads in a magnetic holder (DynaMag-2 Magnet, Life Technologies (part #12321D) for one min. Use a 1.0 mL pipettor to remove the storage buffer and discard it. Add 1.0 mL of the magnetic bead MB Prep Buffer to the tube. Vortex for 1 min to resuspend the beads and then incubate the resuspended beads for 5 mins at room temperature on the bench top, not in the magnet. After the 5 min incubation, place the tube with the beads in the magnet for one min. Pipet off the MB prep buffer and discard. The beads are then washed several times with AB buffer. Add 1.0 mL of AB buffer and vortex to resuspend the beads for 1 min. Place the tube into the magnet for one min and then remove the buffer. Repeat this for a total of three washes. After the washes add 1 mL of Assay Buffer and vortex for 20–30 sec to resuspend the beads. Using an 8-channel pipettor set to 50 μL, the magnetic bead slurry is pipetted into the first four columns (32-wells) of the Catch 2 Omni plate which is then sealed with adhesive foil and stored at 4 °С.

#### Catch 1

After the 3.5 h equilibration of the Catch 0 filter plate it is centrifuged for 2 min at 1000 g to remove unbound proteins. The plate is then washed with 160 μL of SOMAmer-Bead Block solution and removed by vacuum filtration. This is followed by 5 washes with 160 μL of Assay Buffer, the first 4 washes are removed by vacuum and then after the 5^th^ and final wash the plate is centrifuged (2 min, 1000 g) to ensure complete removal of the AB buffer. In this step the biotin moieties of the SOMAmers are being blocked with the SOMAmer-Bead Block solution.

#### Tag & Post-Tag washes

As next step, 11 mL of Assay Buffer is pipetted into the Tag Diluent tube and placed into a 25 °С water bath. Then 110 μL of 100x Tag Reagent is mixed with the 11 mL of Assay Buffer resulting in a 1x Tag Reagent solution. To biotinylate the proteins bound to the SOMAmers, 100 μL of this 1x Tag Reagent is added to each well of the Catch 0 plate, followed by incubation for 5 mins at 25 °С and 850 rpm in the ThermoMixer C. Thereafter, vacuum is applied, the plate is washed twice with 160 μL of Quench Buffer, and then washed 4 times with 160 μL of Assay Buffer. Vacuum filtration is used at all steps except after the final Assay Buffer wash the filter plate is centrifuged for 2 min, 1000 g.

#### Photo-Cleavage of SOMAmers

The next step is to pipet 65 μL of Photo Cleavage Buffer into each well of the Catch 0 Filter plate. The plate is then mixed in the ThermoMixer C for 6 mins at 25 °С and 850 rpm under a UV light source (UV Light Stand, 15 Watt, 365 nm, SomaLogic part #400-00010) followed by rotation of plate 180 degrees in the Thermomixer C and an additional 6 mins of irradiation. These steps cleave the SOMAmers bound to the respective proteins from the beads. Thereafter, the plate is placed on top of the Catch 1 Elution plate and centrifuged for 2 min at 1000 g in order to retrieve the cleaved SOMAmer-protein complexes. Since each sample was set up at 3 different dilutions with their own subset of SOMAmers for the respective set of proteins, each sample will have 3 different eluted SOMAmer-protein solutions. The next step is to combine for each sample these 3 different eluted solutions into a single well.

#### Catch 2

While the photo cleavage is performed, the Catch 2 Omni plate is placed onto the DynaMag-96 side-skirted magnet (Life Technologies, part #12027) that is covered with a custom plate adapter (SomaLogic, part #400-00009) for at least 1 minute. Before the combined eluted SOMAmer-protein eluate from the Catch 1 Elution plate for each sample is added to the Catch 2 plate the magnetic bead supernatant is removed and discarded. The magnetic beads and Catch 1 elution solution and then incubated for 5 mins at 25 °С at 1700 rpm in the ThermoMixer C. After the 5 minutes of mixing 6 μL of MB Block solution is added, followed by a subsequent 2 min incubation on the ThermoMixer C (25 °С, 1700 rpm).

#### High volume washes

The Catch 2 plate is then placed on the magnet for 30 sec and washed twice with 140 μL of Assay Buffer. After removal of this wash step, 200 μL of the Assay Buffer is added and the Omni plate incubated on the ThermoMixer C for 1 min at 25 °С, 1700 rpm.

#### 37 °С temperature controlled washes

The Catch 2 plate is placed on the magnet again for 1 min and the supernatant removed. The beads in the Catch 2 plate are washed 3 times by adding 75 μL of Assay Buffer and incubating for 1 min at 38 °C and 1700 rpm on the ThermoMixer C followed by adding 75 μL of MB Wash Buffer for 2 mins at 38 °C and 1700 rpm. The beads are then separated on the magnet for 30 sec and supernatant removed.

#### High volume washes

The Catch 2 plate is then incubated on the ThermoMixer C for 1 min at 25 °C and 1700 rpm with 200 μL of the same buffer, placed on the magnet for 30 sec and supernatant removed. 140 μL of Assay Buffer is then added twice and the plate incubated for 15 sec on the magnet. This wash step is then removed from the Omni plate.

#### Elution of SOMAmers

As a next step, 75 μL of Elution Buffer is pipetted into each well of the Catch 2 Plate to release the SOMAmers from the SOMAmer-protein complex by incubation for 10 mins at 25 °C, 1700 rpm in the ThermoMixer C. The supernatant containing the eluted SOMAmers is then separated from the beads on the magnet for 30 sec and transferred to the Archive plate and sealed with foil. The eluted SOMAmers can be stored in the Archive plate in a tight fitting ziplock bag at −20 °C for up to a week, or one can proceed immediately to the overnight hybridization step.

#### Hybridization preparation

The next step is the preparation for hybridization of the SOMAmers to complementary DNA strand on the supplied printed microarray slides. The hybridization solution is set-up in a 96-well Omni plate. First, 5 μL of the 10x Slide Block is added to each well of the Omni Hybridization plate. The Slide Block contains spiked in hybridization controls at low, medium and high concentration. This is combined with 20 μL of the Catch 2 eluate from the Archive plate and 25 μL of the Agilent 2x Hybridization Buffer. The Hybridization solution should be well mixed by pipetting up and down 15–20x. Do not pipet so hard that large bubbles are introduced into the mixture. The Hybridization Plate is covered with foil centrifuged for 2 min at 1000 g.

#### Hybridization

Slides are loaded using the Agilent Hybridization Chamber SureHyb Enabled, Stainless (Part #G2534A). For the 32 samples, four printed microarray slides are needed. Five of the Agilent Hybridization Chambers are required as the fifth chamber is used to aid is consistent dropping of the microarray slide onto the gasket slides. Each gasket well of the 8 arrays per slide (Agilent, Part #G2534-60016) is loaded with 40 μL of the hybridization solution from the Hybridization Omni plate. The printed microarray slide is then dropped onto the top of the sample loaded gasket slide using the ends of the chamber as recommended by Agilent. The four sealed and tightened Hybridization Chambers are placed into a hybridization oven (Agilent, Part #G2545A) for a 19 h incubation at 55 °C with a 20 rpm rotation.

#### Array processing and scanning

After hybridization the microarray sandwich is disassembled in a dish (ThermoFisher, Part #122) containing Agilent Slide Wash 1. The array itself is then put into the slide rack of another dish (ThermoFisher, Part #121) with Wash Buffer 1 and incubated for 5 mins at room temperature with a stir bar stirring (250 rpm). The four slides in the slide rack are then quickly transferred to a second dish with Agilent Wash Buffer 2 solution from the 37 °C oven which is placed on a hot plate at 80 °C for 5 mins with stirring (250 rpm). The slide rack is slowly removed from Wash Buffer 2 (10–15 seconds) and dropped into a third staining dish containing 100% acetonitrile at room temperature for 5 mins, stirring at 250 rpm. The slide rack containing the now washed slides are then removed and are ready for scanning. The microarrays are analyzed using an Agilent SureScan Microarray Scanner (Part #G4900DA). The raw data is assembled by SomaLogic into an ADAT^26^ file (HMS-16-007.20160218.adat). The ADAT file is then subjected to Hybridization Control Normalization (resulting file HMS-16-007.HybNorm.20160218.adat), Median Signal Normalization (resulting file HMS-16-007.HybNorm.MedNorm.20160218.adat), and Calibration Normalization (resulting file HMS-16-007.HybNorm.MedNorm.Cal.20160218.adat) steps according to the standard quality control protocols at SomaLogic. All samples passed the established quality control criteria. The resulting ADAT file (HMS-16-007.HybNorm.MedNorm.Cal.20160218.adat and HMS-16-007.HybNorm.MedNorm.Cal.20160218.xls) is ready for further analysis. An example of downstream statistical analysis of the dataset using *readat*^26^ Bioconductor package is given in the supplemental R script^27^. A metadata file (patients_metadata.txt) links patient designations with demographic and clinical data presented in the Tables 1 and 2.

## Data Records

All data resulted from this study has been submitted to the *Figshare* repository^27^.

***HMS-16-007.20160218.adat*** - raw SomaScan dataset presented in adat format^26^.

***HMS-16-007_SQS_20160218.pdf*** - technical validation report on the dataset.

***HMS-16-007.HybNorm.20160218.adat*** - SomaScan dataset after hybridization control normalization presented in *adat* format^26^.

***HMS-16-007.HybNorm.MedNorm.20160218.adat*** - SomaScan dataset after hybridization control normalization and median signal normalization presented in *adat* format^26^.

***HMS-16-007.HybNorm.MedNorm.Cal.20160218.adat*** - SomaScan dataset after hybridization control normalization, median signal normalization, and calibration presented in *adat* format^26^.

***HMS-16-007.HybNorm.MedNorm.Cal.20160218.xls*** - SomaScan dataset after hybridization control normalization, median signal normalization, and calibration presented in Microsoft Excel Spreadsheet format.

***Patients_metadata.txt*** – metadata file containing patients’ demographic and clinical information presented in tab-delimited text format. Metadata is linked to records in the SomaScan dataset via ‘SampleType’ column.

## Technical Validation

All SomaScan control samples for technical validation procedures were set up and run according to SomaLogic recommendations. All technical validation procedures were carried out according to SomaLogic algorithms at SomaLogic. SomaLogic Quality Report (HMS-16-007_SQS_20160218.pdf) has been submitted to *Figshare* repository^27^.

## Supplementary information

Supplemental table S1

## Data Availability

**Readat (version 1.4.0)** script is available on Bioconductor (www.bioconductor.org). **SciData_R_script.R** – this supplemental script is given as an example of a downstream statistical analysis of the HMS-16-007.HybNorm.MedNorm.Cal.20160218.adat dataset^27^.
